# An experimental simulation model to assess wear of the porcine patellofemoral joint

**DOI:** 10.1371/journal.pone.0250077

**Published:** 2021-04-26

**Authors:** Raelene M. Cowie, Philippa Bowland, Divya Baji, Hazel L. Fermor, Eileen Ingham, John Fisher, Louise M. Jennings

**Affiliations:** 1 Institute of Medical and Biological Engineering, School of Mechanical Engineering, University of Leeds, Leeds, United Kingdom; 2 Faculty of Biological Sciences, Institute of Medical and Biological Engineering, University of Leeds, Leeds, United Kingdom; Ohio State University, UNITED STATES

## Abstract

A range of surgical techniques and osteochondral interventions have been developed for early stage chondral/osteochondral repair interventions in the knee however, methods for functional, pre-clinical assessment of these therapies are limited. In this study, a method for simulating physiological loading and motion in the porcine patellofemoral joint was developed using a 6-axis simulator. As an example of how the method can be used, the influence of surgical positioning of osteochondral allografts in the patella on cartilage wear, deformation and damage and graft stability was investigated in this porcine patellofemoral joint model. The functional performance of allografts implanted either optimally (flush with the cartilage surface) or 1 mm proud of the cartilage surface was compared to a positive control (stainless steel pin implanted 1 mm proud of the cartilage surface), a negative control (no intervention) and a defect model. Allografts implanted flush with the surrounding cartilage could restore the articulating surface of the patella resulting in low wear, damage and deformation of the opposing cartilage surface, similar to that of the negative control group. Implanting the graft proud of the patella surface resulted in cartilage lesions on the femoral trochlea (ICRS grade 2) and a cartilage volume difference of 2.0 ± 3.9 mm^3^; the positive controls resulted in more severe lesions, a higher volume difference (14.2 ± 7.4 mm^3^) which in some cases exposed subchondral bone (ICRS grade 4). Defects in the patella caused deformation of the opposing cartilage surface. All grafts implanted in the patella subsided over the duration of the study. This study demonstrated a method that can be used to evaluate osteochondral repair strategies in the patellofemoral joint applying physiological loading and motions.

## Introduction

Chondral and osteochondral lesions in the patellofemoral joint (PFJ) are common, however reporting of the prevalence of these lesions is highly variable. Widuchowski et al. showed 36% of all lesions in the knee occurred in the PFJ, more common than the medial femoral condyle [1, 2], with 40% of these lesions graded 3 or 4 on the Outerbridge classification [3]. Other studies however have shown a lower proportion of osteochondral lesions in the PFJ at either ~20% [4] or ~10% [5, 6]. Whilst many lesions may be asymptomatic [2], osteoarthritis (OA) in the PFJ is often a precursor to OA in the tibiofemoral joint (TFJ), therefore effective treatment strategies must be developed for their management [7]. Cartilage lesions within the patellofemoral joint occur more frequently on the patella, particularly on its medial aspect, rather than on the trochlear groove [1, 2], damage may occur as a result of knee injuries including ACL rupture and dislocation or due to biomechanical abnormalities including patella instability [8]. Isolated PFJ lesions most commonly occur in young females and it is this subgroup who are most likely to have the most severe degeneration requiring treatment [7]. Conservative management involving physical therapy and analgesics is preferred for the majority of PFJ chondral lesions however, a number of surgical interventions have been developed to restore the articulating surfaces. Techniques such as debridement, microfracture, autologous osteochondral grafting (mosaicplasty), cell based therapies such as autologous chondrocyte implantation (ACI) or matrix-assisted ACI (MACI) and unicompartmental arthroplasty have been successfully used in the treatment of chondral/osteochondral lesions in the TFJ [9]. However, when applied to the PFJ, surgical outcomes have often been less successful and the reasons for this are unclear [8, 10, 11].

To better understand how surgical technique can influence the function of interventions in the patella immediately post-surgery, there is a need for pre-clinical investigations. *In vivo* models, primarily ovine studies have been used, however they are costly and time-consuming. The development of an *in vitro* model that replicates the loading and motion of the joint is warranted for use as a pre-clinical assessment tool prior to *in vivo* studies reducing the use of living animals. An experimental simulation model of the TFJ has been developed [12–14] but the geometry, forces and kinematics in the PFJ require the evolution of additional simulation systems. Experimental pre-clinical simulation models reported to date have used porcine tissue [12–15], and whilst it is recognised that the use of cadaveric human donor tissue would better replicate the host environment, animal tissue can be used to help establish methods which can later be transferred to human joints. When determining the tissue source, the joint size, cartilage and trabecular bone thickness have been considered and porcine tissue most closely matched the human TFJ and PFJ [16]. Use of tissue from healthy animals that is readily available gives high quality tissue and reduces inter-specimen variability. Further, the use of healthy/non-pathological tissue with a defined focal lesion may be more representative of the tissue in which an early intervention such as an osteochondral graft would be considered.

The aim of this study was to develop an experimental simulation model to pre-clinically assess and predict the wear of osteochondral allografts in the natural PFJ. The method used a porcine PFJ mounted in a knee simulator with loading and motion to replicate a walking gait cycle, this experimental simulation model was then verified by investigating the wear of osteochondral allografts (OCGs) which have been used clinically in the PFJ to repair small focal lesions [7, 8]. Specifically, by measuring the cartilage geometry, which is a combination of the loss of material (wear) and permanent deformation, the method assessed a combination of the cartilage wear, deformation and damage in addition to graft stability following functional simulation. The effect of surgical precision on graft implantation was explored initially through assessment of grafts positioned proud of the patella surface. It was important for the method verification to determine whether it was possible to differentiate between different experimental groups.

## Materials and methods

### Materials/animal tissue

Porcine PFJs were obtained from the right hind legs of Large White pigs aged either 4–6 or 12 months old (John Penny & Sons, Leeds, UK), within 24 hrs of slaughter. The two ages were used to give a model of different bone properties such as elastic yield and modulus. Tissue samples were kept hydrated throughout preparation using phosphate buffered saline (PBS; MP Biomedicals LLC, UK) and stored at -20°C. Prior to testing, samples were thawed at room temperature.

### Simulation method development

**Fig 1:**
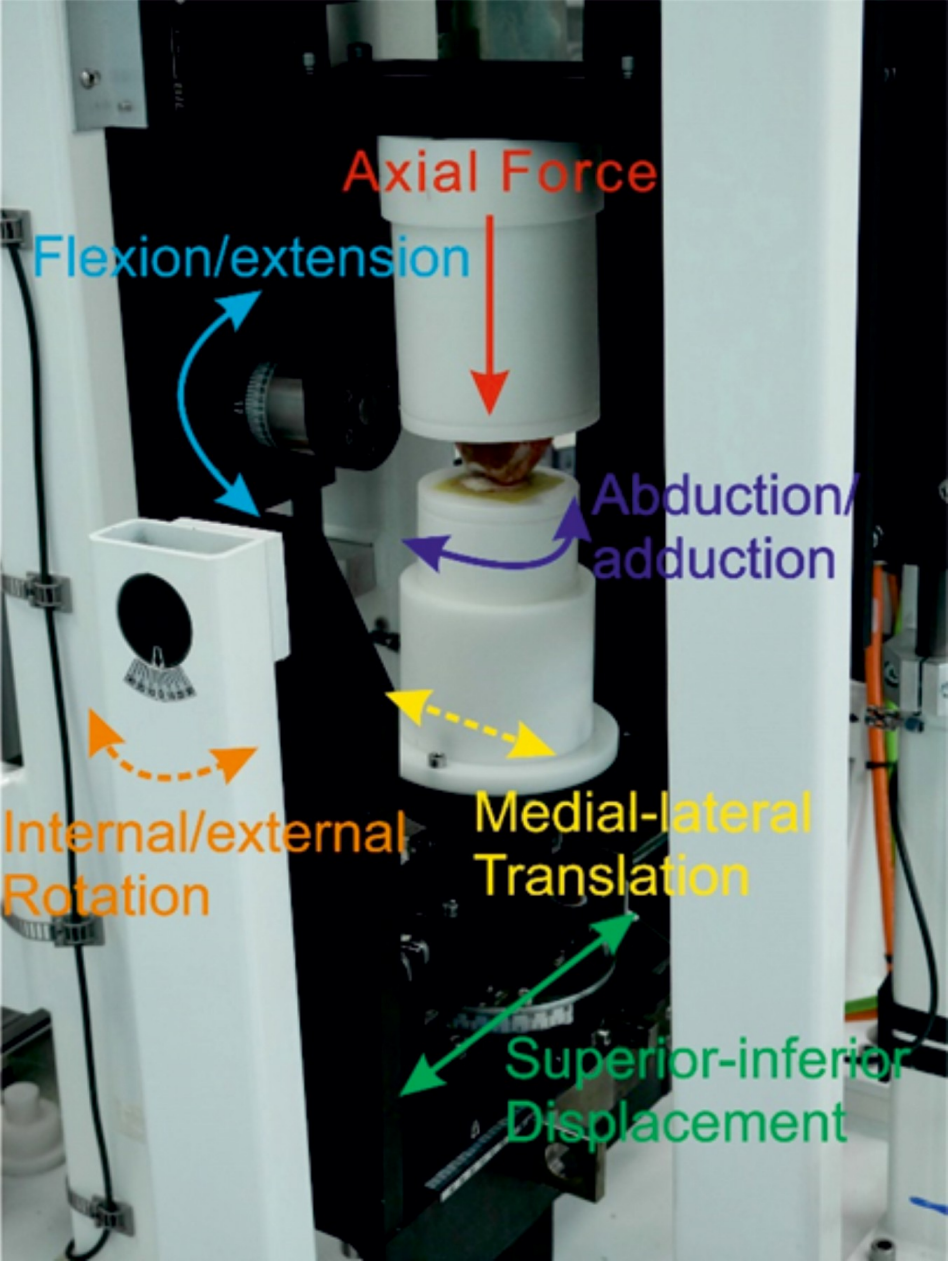
A porcine PFJ mounted in a single station knee simulator. The solid arrows represent controlled/driven axes (AF, FE, AA, SI), the dashed lines represent either uncontrolled axes (M-L) or axes constrained using springs (IE). During the simulation, the joint was enclosed within a gaiter filled with 25% (v/v) bovine serum in PBS.

The simulation was carried out by adapting a single station, six-axis electromechanical simulator (Simulation Solutions, UK) initially designed for TFJ simulation (Fig 1). The simulator has 4 controlled axes of motion, axial force (AF), flexion extension (FE), superior-inferior displacement (SI) and abduction/adduction rotation (AA). The AF was force controlled with the other driven axes displacement controlled. In addition, the patella was allowed to translate freely in the medial-lateral direction (ML) to track the trochlear groove and the patella tilt (IE rotation) was constrained using springs (1.61 N/mm). Constraint of patella tilt was required as during method development, some samples, especially those in which the trochlear groove was less clearly defined at its most superior aspect, were prone to dislocation. The spring rate was optimised to minimise constraint of the patella whilst preventing dislocation. The FE and AF were applied through the femur, all other motions acted on the patella.

**Fig 2:**
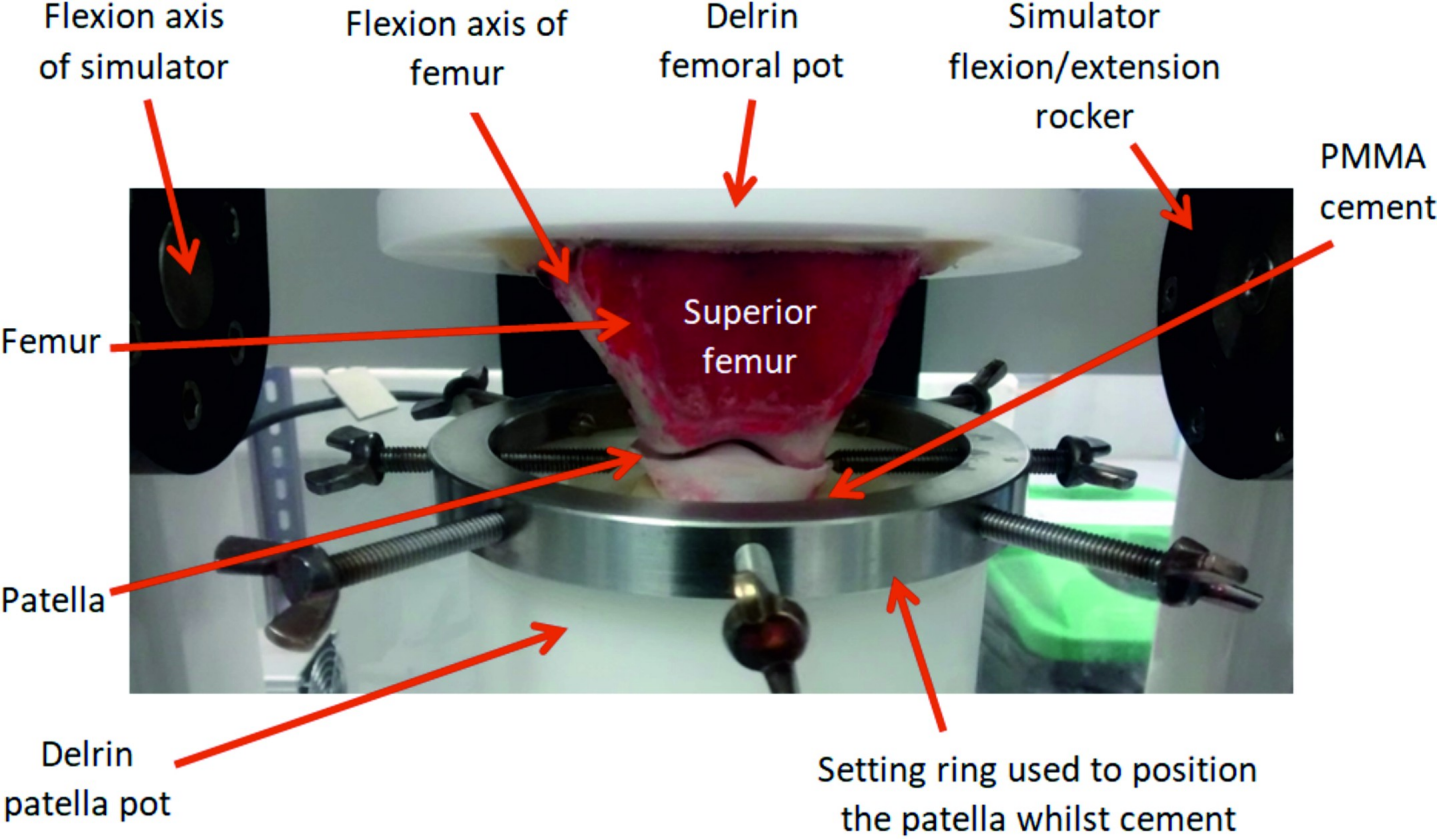
A natural porcine PFJ cemented into custom Delrin fixtures. The centre of rotation of the trochlear groove (flexion axis of the femoral trochlea) aligned with the flexion axis of the simulator, the photograph was taken during cementing of the patella carried out within the simulator with a setting ring used to position the patella whilst the PMMA cement cured.

To mount the porcine PFJ in the simulator, all soft tissue surrounding the knee joint including the capsule and tendons were dissected ensuring no damage occurred to the cartilage and the patella was separated from the femur. Removing the soft tissue is consistent with previous studies of the TFJ [12, 13] and optimised access to the articulating surfaces of the joint for both the introduction of the intervention into the joint and also analysis of the cartilage surfaces. The relative motion of the patella and femoral trochlea were then replicated using the simulator [14]. To maintain consistency of set-up between samples, the positioning of the flexion axis of the femoral trochlea was critical. The centre of rotation of the femoral trochlea was located using a templating method, previously described by McCann et al. [17] to determine the radius of the medial and lateral edges of the trochlear groove at 0° flexion. The femur was then cemented into custom fixtures using poly methyl methacrylate (PMMA) cement (WHW Plastics, UK) with the centre of rotation of the femoral trochlea aligned with the flexion axis of the simulator. To position the patella, first, the femoral trochlea was moved into full extension and the patella fixture was fixed in its neutral position (0 mm SI displacement, 0° AA rotation, 0 mm ML displacement and 0° IE rotation), PMMA cement with a dough-like consistency was placed in the patella fixture and the patella manipulated to align the surface of the patella with the IE axis of the simulator and to maximise the contact between the patella and trochlear groove. A setting ring held the patella in position whilst the cement cured (Fig 2). Throughout this process, the cartilage surfaces were hydrated by spraying with PBS.

**Fig 3:**
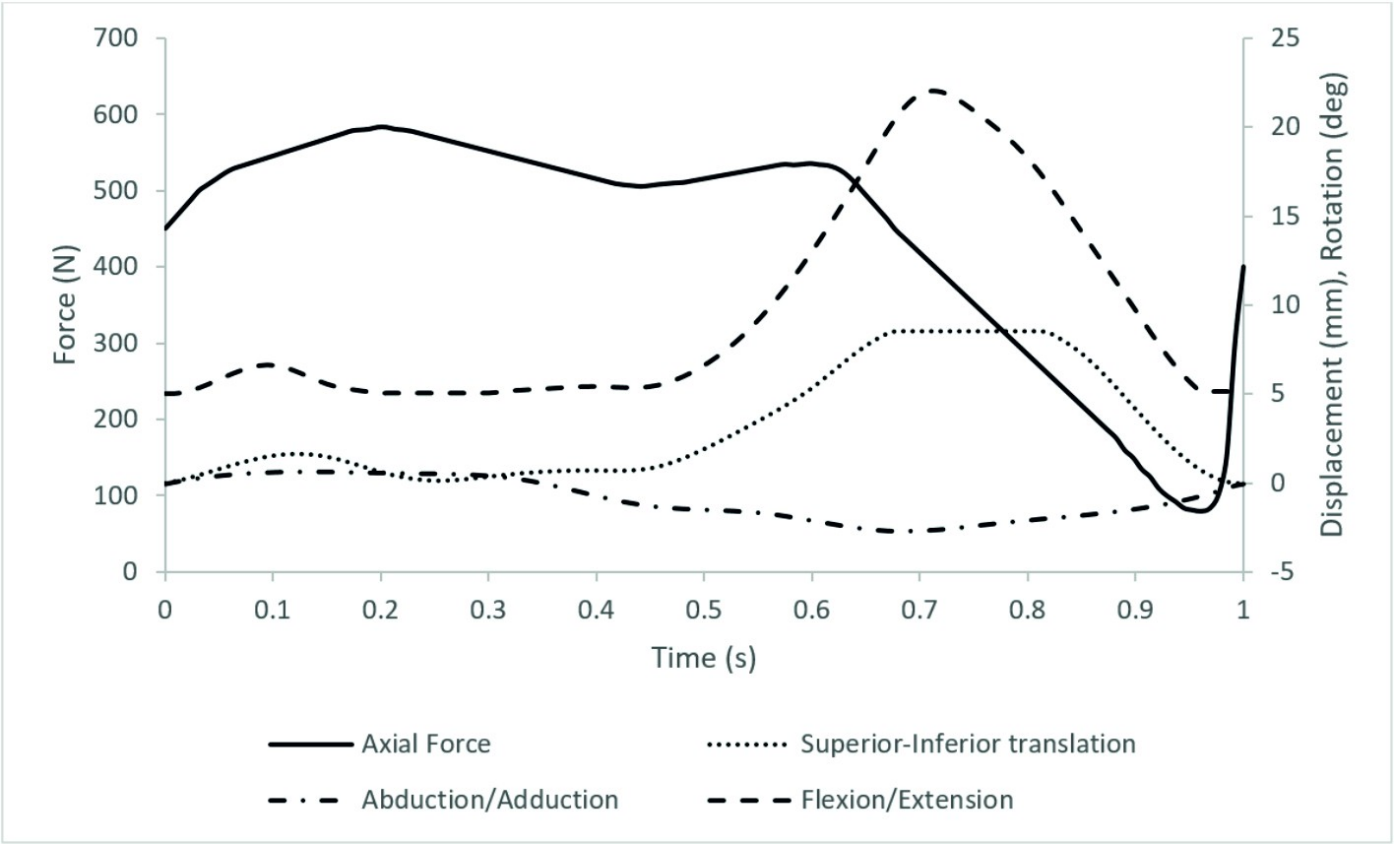
The simulation input profiles showing axial force (N), superior-inferior translation (mm), abduction/adduction (deg) and flexion/extension (deg). Medial-Lateral translation was passive and Internal/External rotation constrained using springs.

The input profiles used were adapted from previous work on the artificial PFJ by Maiti et al. and based on a walking gait [18, 19], the magnitude of the loading and motions were scaled similar to previous studies using porcine tissue to accommodate the smaller porcine joint (Fig 3). During the set-up process, Microset 101FF (Microset Products Ltd., Hinckley, UK), a silicone replicating compound was applied to the patella and used to ensure there was contact between the patella and the trochlear groove throughout the gait cycle [12, 20].

### Experimental design

The experimental groups are summarised in Table 1. All interventions were implanted into the centre of the medial aspect of the patella which is the most common site for focal chondral defects in the PFJ [2] and the surgical outcomes of grafts implanted in this region are often poor [21]. The experimental design of the study, previously described by Bowland et al. [13] involved initially running each experimental group (excluding the negative control), for 15 minutes (900 cycles) to check the stability of the joint before introducing the intervention into the patella and running the simulation of the joint for an additional 3 hours (10800 cycles); in addition, there were full negative and positive control groups to verify the method development. In the negative control group, the natural PFJ was run without any intervention for 3 hours [13]. In the positive control group, a stainless steel (SS) pin was implanted proud of the articulating surface. The purpose of the positive control group was to demonstrate that the simulation model was sensitive enough to detect wear, deformation and damage. All simulations were carried out using 25% (v/v) bovine serum in PBS as a lubricant with the cartilage surfaces submerged in the lubricant throughout.

**Table 1:** The experimental groups investigated, all groups were studied for 3 hours of simulation at 1Hz (10,800 cycles) in a single station knee simulator.

Experimental group	Description	Age of animals	Number of samples
Negative control	Articulating surfaces intact, no intervention	4–6 months	6
Positive control (stainless steel pin 1mm proud)	6.5 mm diameter stainless steel pin implanted 1mm proud of cartilage surface	4–6 months	6
Cartilage defects	6 mm diameter defect created to expose subchondral bone	4–6 months	5
Osteochondral allograft	6.5 mm diameter osteochondral graft from porcine tissue implanted flush with the cartilage surface	4–6 months	5
Osteochondral allograft 1mm proud	6.5 mm diameter osteochondral graft from porcine tissue implanted 1mm proud of the cartilage surface	4–6 months	5
Positive control (stainless steel pin 1mm proud)	6.5 mm diameter stainless steel pin implanted 1mm proud of cartilage surface	12 months	5

### Osteochondral grafting

The 6.5 mm diameter Accufex mosaicplasty kit (Smith and Nephew, MA, USA) was used for harvesting the osteochondral grafts and recipient site creation. Osteochondral allografts were harvested from healthy porcine knees, and a flat base was created using a file, the flush grafts were 10 mm in length and the proud grafts 11 mm. Recipient sites were created with a depth of 10 mm consistent for all experimental groups using a drill and a flat base created using a tamp. Dilation of the recipient site was not carried out consistent with previous experimental studies of osteochondral grafting in porcine tissue which showed a discontinuity between the graft and host tissue following dilation [13, 22]. For the defect group, a biopsy punch was used to create a 6 mm diameter full thickness cartilage defect which exposed the underlying subchondral bone. For the positive controls, stainless steel pins of 6.5 mmm diameter and 11 mm length with a radius of curvature of 100 mm, a polished surface (Ra <0.01 μm) and radiused edge were implanted so they sat proud of the cartilage surface by 1 mm.

### Grading of cartilage lesions

To assess the change in the articulating surfaces following the simulation, two methods were used. Firstly, a cartilage scoring system based on the International Cartilage Regeneration and Joint Preservation Society (ICRS) cartilage evaluation grading system for cartilage lesions was used [23], with two independent researchers grading each sample. Each patella was divided into 9 distinct regions with the region containing the intervention (medial-central) excluded from analysis; the femoral trochlea was divided into 3 regions, medial, central and lateral. The most severe cartilage lesion in each region was scored from 1 to 4 with normal, healthy cartilage scored 0 and severely abnormal cartilage with exposed subchondral bone graded 4. The maximum score with damage through to subchondral bone in all regions was therefore 32 and 12 for the patella and femoral trochlea respectively.

### Assessment of graft stability and wear, damage and deformation volume

The cartilage surfaces were also assessed quantitatively from silicone replicas of the articulating surfaces taken using Accutrans AB replicating compound (Coltene Whaledorf AG, Switzerland). Replicas were taken following graft implantation and at the study conclusion and analysed using an Alicona IF G5 optical profilometer (Graz, Austria) with 5X magnification. As a measure of graft stability, the graft height was assessed pre- and post-simulation from the replicas by taking an average of 8 step height measurements between the top of the graft and surrounding tissue. When the graft was proud of the surface of the patella, the measurement was positive, negative values indicated graft subsidence below the patella surface. On the femoral trochlea, where possible, the cartilage wear, deformation and damage was assessed in terms of the volume change as previously described by Bowland et al. [13].

### Data analysis

For the non-parametric data, in this case the cartilage grading, the mean cartilage score with standard deviation (SD) was determined. For the parametric data, the mean graft height (mm) and wear, deformation and damage volume (mm^3^) was calculated with 95% confidence limits (CL). For all data, statistical analysis carried out in IBM SPSS Statistics for Windows, Version 26 (Armonk, NY, US). Having carried out a variance test, the cartilage grading score and wear, deformation and damage volume was analysed using a Kruskal Wallis test, post-hoc testing was performed using the Dunn-Bonferroni approach. Significance was taken at p<0.05.

The data associated with this article are openly available through the University of Leeds data repository [24].

## Results

### Optimisation of the simulation system

Following setup of the joint, Microset 121FF was applied to the trochlea and the simulation run to determine the contact between the trochlear groove and patella. The setup was considered to have been optimised when contact occurred over the width of the trochlea between its mid and superior aspects and over the whole of the patella. The constraint of the patella tilt was also tuned by investigating springs of different spring rate with the aim of minimising the applied constraint whilst preventing dislocation of the joint. In this simulation system, a spring tension of 1.61 N/mm prevented dislocation whilst allowing the patella to tilt.

### Cartilage wear, deformation and damage following functional simulation

**Fig 4:**
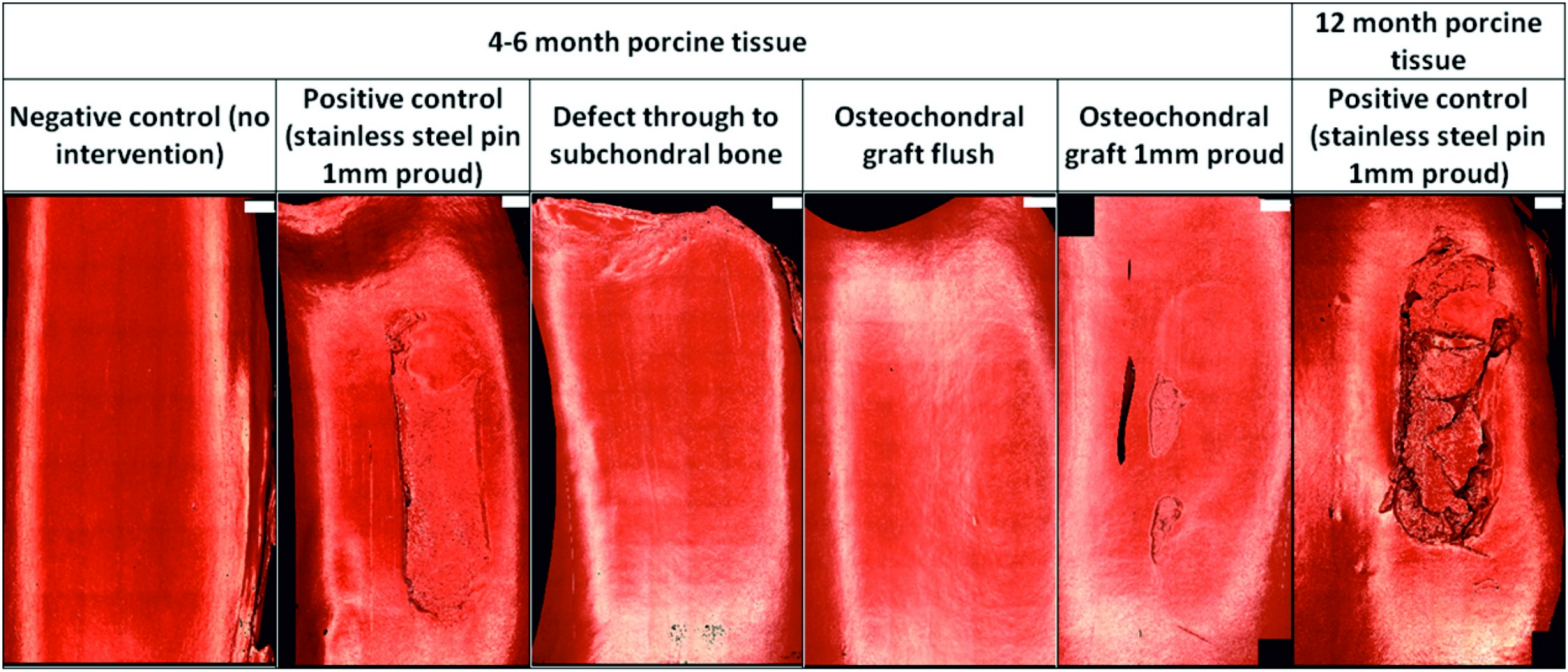
Representative images of Accutrans replicas of the wear scar on femoral trochlea following 3 hours (10,800 cycles) simulation. Images taken using an Alicona IF G5 optical profiler with 5X magnification. Scale bars shown in white at the top right of each image represent 2mm.

**Fig 5:**
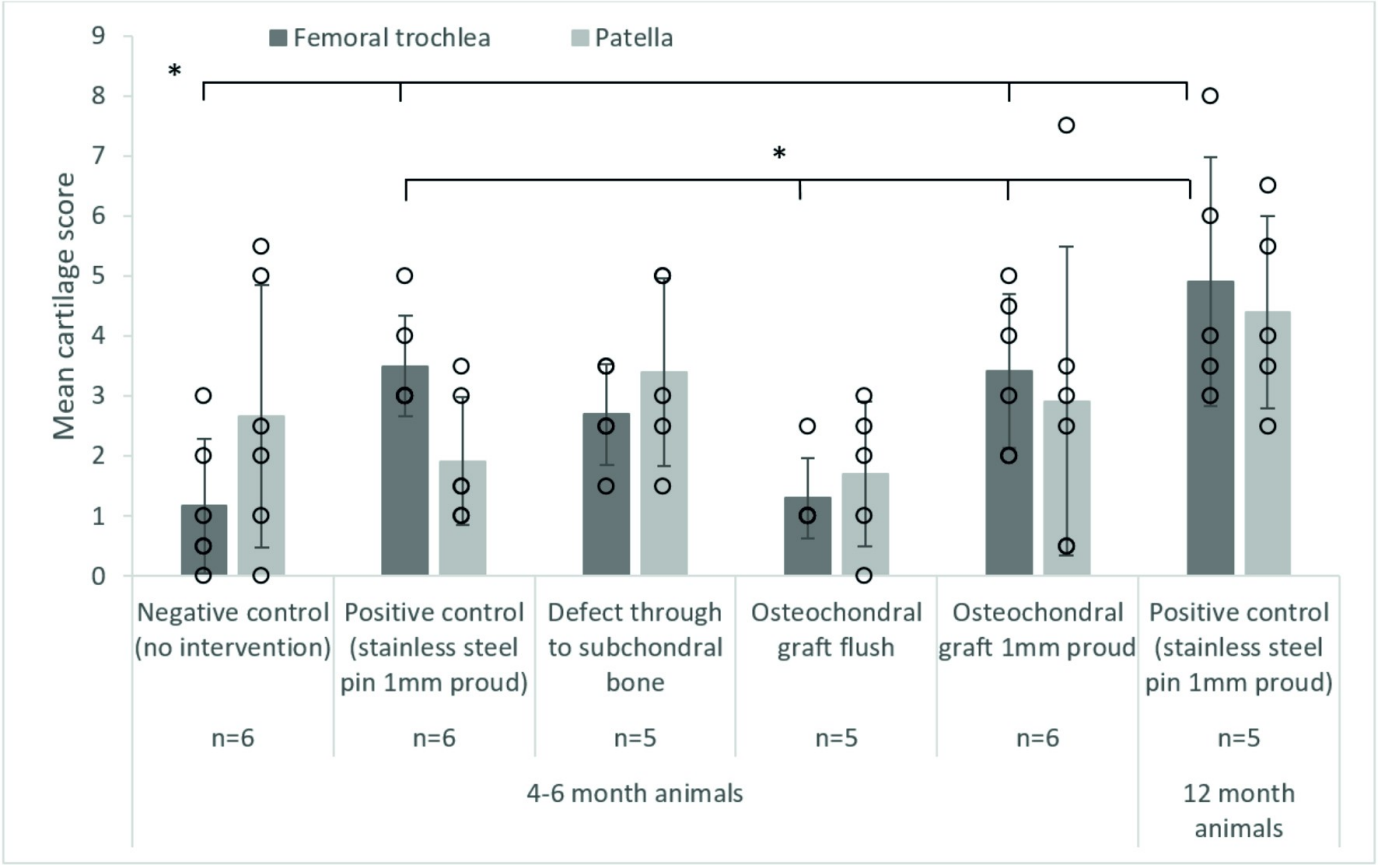
Mean (± SD) cartilage score on the patella and femoral trochlea after 3 hours (10,800 cycles) wear simulation, * denotes p<0.05, there were no significant differences between patella groups. The data points from all experimental groups have been overlaid as a dot plot.

Representative images of Accutrans replicas taken from the surface of the femoral trochlea from all experimental groups at the conclusion of the study are shown in Fig 4. Six samples were tested for 3 hours without an intervention (negative controls), dislocation did not occur in any samples however, some isolated scratches were visible on both the patella and femoral trochlea. The mean cartilage ICRS scores were 2.6±2.2 and 1.2±1.1 for the patella and femoral trochlea respectively (Fig 5), all lesions were classed as grade 1.

Cartilage scores on the femoral trochlea of the negative control groups (1.2±1.1) were similar to the OCGs implanted flush with the cartilage surface (1.1±0.8); lesions were also graded 1. Introduction of a cartilage defect into the patella resulted in a circular deformation on the opposing femoral trochlea, when the samples were left unloaded for 24 hours following simulation, this deformation was no longer visible. Around the cartilage defect site, there was evidence of cartilage damage (grade 1/2) and on the femoral trochlea, grade 2 lesions were observed in some samples. The mean cartilage score on the femoral trochlea of the cartilage defect group was therefore higher than that for the negative controls and OCGs, 2.3±0.8.

When either stainless steel pins or allografts were implanted proud of the surface of the patella, cartilage damage was visible on the opposing femoral trochlea. For OCGs positioned proud of the articulating surfaces, lesions on the femoral trochlea were predominantly ICRS grade 2/3, with a mean score of 3.4±1.4. Implanting a stainless steel pin in the patella (as a positive control) resulted in deeper femoral trochlea lesions, in some cases exposing subchondral bone (ICRS grade 3/4) however, the mean cartilage score was similar to the OCGs implanted proud, 3.5±0.8. In more mature porcine tissue, there was evidence of chondral delamination in which cartilage had been pulled away from the underlying bone; chondral delamination did not occur in any studies using younger porcine tissue. Damage to the femoral trochlea was primarily confined to a single region where the femoral trochlea opposed the intervention and statistical analysis of the cartilage scores using a Kruskal Wallis test showed a significant difference (p = 0.001) between the experimental groups. For the patella, there was no significant difference (p = 0.179) between the experimental groups, lesions were primarily graded 1 and no cartilage lesions classed greater than 2.

**Fig 6:**
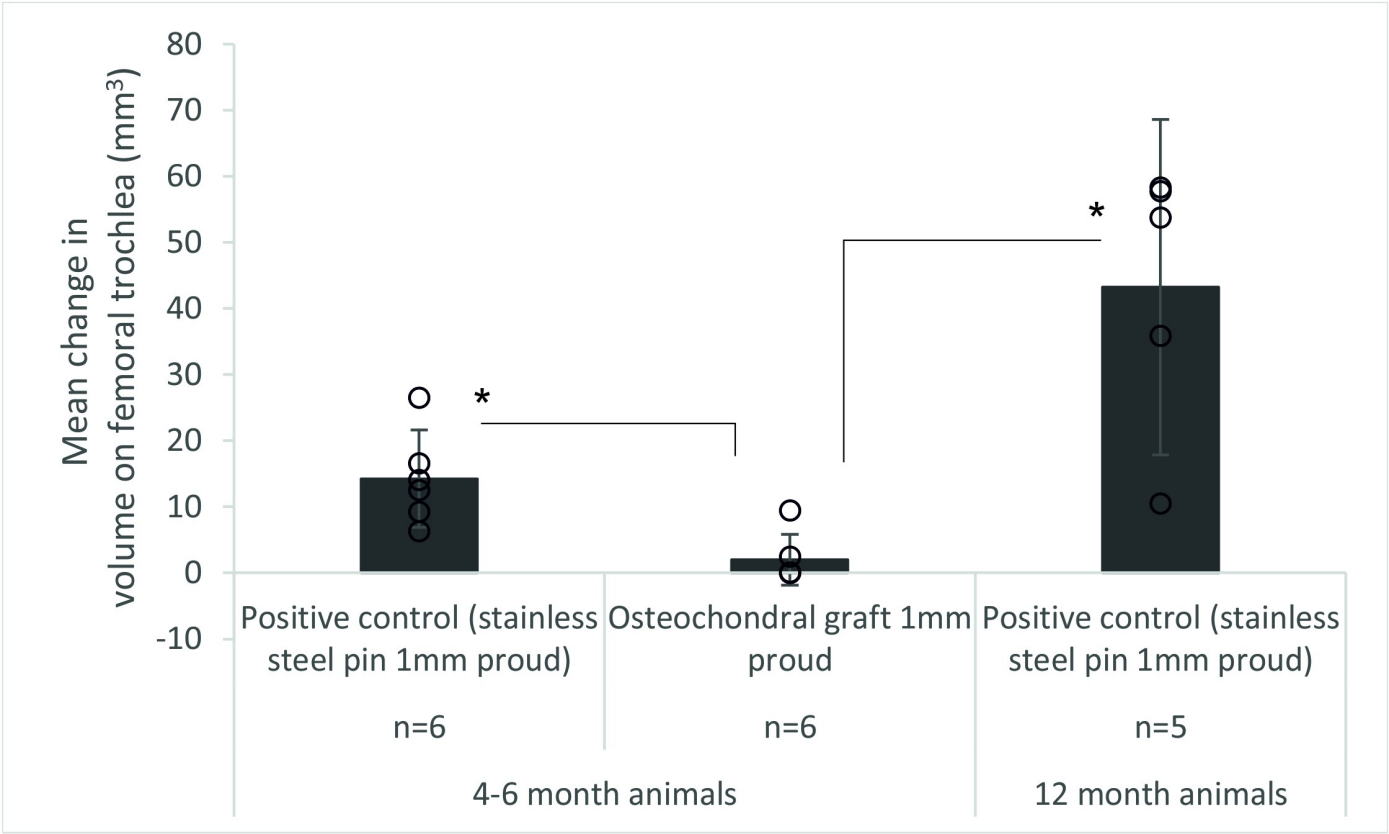
Mean (±95% CL) volume (mm^3^) of wear, deformation and damage in the cartilage on the femoral trochlea after 3 hours (10,800 cycles) simulation of grafts positioned proud of the patella, * denotes p<0.05. **6.** The data points from all experimental groups have been overlaid as a dot plot, for the osteochondral graft group, the wear volume was not measurable on some samples.

The volumetric wear, deformation and damage in the femoral trochlea was assessed quantitatively from replicas of the cartilage surfaces (Fig 6). For the negative control, defect and OCG flush groups, the wear, deformation and damage was below a threshold measurable using the optical profilometry technique. The change in volume was highest for the more mature porcine tissue with a positive control stainless steel pin implanted proud of the cartilage surface; for OCGs implanted proud of the patella surface, the mean wear volume was low and for some samples, the region of damage was too poorly defined to assess. There was a significant difference (p = 0.005) in mean change in volume of cartilage on the femoral trochlea between the osteochondral allograft 1 mm proud group and the positive control groups with stainless steel pins implanted 1 mm proud for both 4–6 month and 12 month animals (Fig 6), when the depth of the wear scar was assessed, similar trends were observed (p = 0.005) however, the area of the wear scar was similar (p = 0.692) for all experimental groups. Further analysis of the wear scars has been presented in the Leeds University data repository [24].

### Graft stability during functional simulation

**Fig 7:**
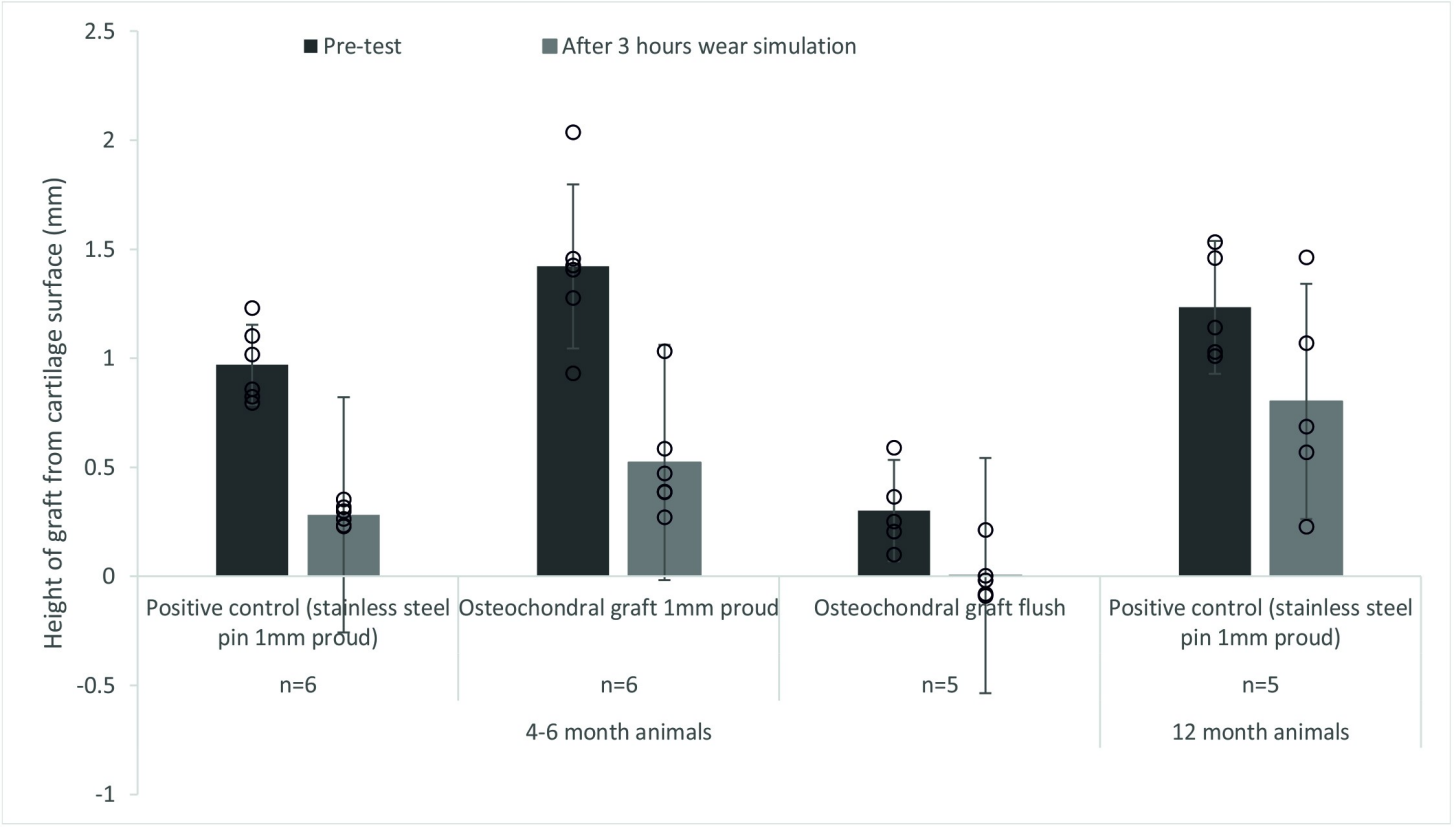
Mean (±95% CL) protrusion of the pin/osteochondral graft from the cartilage surface (mm). A comparison between the protrusion of the pin prior to the start of the study and after 3 hours (10,800 cycles) of simulation under a walking gait cycle. The data points from all experimental groups have been overlaid as a dot plot.

After 3 hours wear simulation, there was evidence of graft subsidence in all experimental groups (Fig 7). In the 4–6 month old porcine tissue, stainless steel positive control pins and OCGs positioned proud of the surface of the patella subsided similarly with a decrease in mean graft height of 69.9% and 64.1% for the stainless steel positive control pins and OCG respectively. In some cases, OCG’s initially implanted flush with the surface subsided below the articulating surface of the cartilage. For 12 month old porcine tissue, mean subsidence of the stainless steel positive control pins was lower (36.6%) than for 4–6 month old porcine tissue (69.9%).

## Discussion

This is the first study to establish an experimental simulation method for predicting wear, damage and deformation of osteochondral allografts in the natural porcine PFJ under physiological loading and motion. Having established a reproducible simulation method which gave repeatable contact between the samples without dislocation, it was then used to start to investigate the influence of surgical positioning of osteochondral allografts in the patella. The wear, deformation and damage of the cartilage surfaces was determined through grading of cartilage lesions using a modified ICRS grading system and by measuring the volume of cartilage wear, deformation and damage on the femoral trochlea. The stability of the graft in the patella after being subjected to continuous loading and motion was also assessed.

### Cartilage wear, deformation and damage following functional simulation

Prior to assessing the performance of OCGs, a negative control group was investigated, in which the PFJs were mounted and run for 3 hours to determine the cartilage wear, deformation and damage in this natural simulation system. After 3 hours simulation, no cartilage deformation was visible however, on some samples, isolated, fine scratches on both the femoral trochlea and the patella running the length of the cartilage surface and covering multiple regions in the cartilage analysis were apparent. This led to a relatively high mean cartilage grading score for both the patella and femoral trochlea however, the lesions were scored no higher than grade 1 and it is hypothesised that fragments of bone released from the cut ends of the femur may have contributed to the scratches. As the setup of the joints was further optimised, this damage mode became less apparent, especially in the experimental groups with proud grafts where the contact area was lower.

When defects were created in the cartilage, after 3 hours of simulation, deterioration of the edge of the defect site was evident and cartilage deformation was visible on the opposing femoral trochlea, resulting in a mean femoral trochlea cartilage grading score higher than that of the negative controls. Unloading the samples for 24 hours resulted in the deformation becoming less visible. This highlighted a limitation with the experimental approach which applied continuous loading and motion to the cartilage over a period of 3 hours without rest periods for the cartilage to rehydrate/recover. The use of a perfectly circular defect may have questionable clinical significance and may be more representative of a recessed graft however, the defect was of similar size to subsequent interventions and followed a similar rationale to Bowland et al. [13].

When osteochondral allografts were implanted flush with the articulating surfaces, the damage scores on the patella and femoral trochlea were similar to that of the negative controls and lower than that of the defects which demonstrated that well positioned allografts can be used to reconstruct the articulating surfaces.

Grafts positioned proud of the surface of the patella produced measurable wear, deformation and damage of the cartilage in the trochlear groove. It is a limitation of the modified ICRS scoring technique used that the OCG proud and positive control in 4–6 month old animal tissue could not be differentiated when the damage was evidently greater on the positive control samples however, volumetric analysis of this cartilage damage allowed the experimental groups to be clearly differentiated. The wear, deformation and damage volume for the positive controls in young animal tissue was >5 times that of the allografts positioned proud and the region of damage was less clearly defined for the allograft group. Bowland et al. showed similar trends in the TFJ with stainless steel pins positioned proud of the surface creating a greater volume of wear, deformation and damage than OCGs positioned proud [13]. The wear, deformation and damage volume was highest in the experimental group with older animals where cartilage delamination was also evident however, it is not known whether the age of the animal or the improved graft stability and reduced subsidence had a greater influence on the wear, damage and deformation volume. The technique could however differentiate between flush and proud OCGs and highlights the importance of restoring the congruent articulating surfaces when implanting OCGs to minimise cartilage wear [25–28].

### Graft stability during functional simulation

In this study, subsidence of the OCG (whether a stainless steel pin or an allograft) occurred in all samples. Subsidence of OCGs below the articulating surface can result in inferior biomechanics and healing encouraging the formation of fibrocartilage, which can change the contact pressure within the joint [25, 29, 30]. In 4–6 month old animal tissue, subsidence was similar irrespective of whether a high modulus (stainless steel pin) or lower modulus (OCG) graft was used. However, using tissue from older animals with a higher bone mineral density [31] decreased graft subsidence. In these studies, the modulus and the geometry of the graft was kept constant by using a stainless steel pin which suggests that bone modulus and skeletal maturity particularly of the recipient site influences graft stability [22]. In similar studies of the TFJ using porcine tissue age 4–6 months, grafts of the same length, implanted using a similar technique, graft subsidence was not reported [13]. It is not known whether this was due to differences in the bone properties in the host site or the simulation of the different joints. The result was unexpected as in humans, the bone mineral density and modulus of the bone in the patella is comparable to that of the condyles [32–34] however, there is limited data relating to bone properties in porcine tissue and, differences in the biomechanics of the porcine knee joint compared to a human joint may influence the density and stiffness of bone. Further studies investigating how graft stability and tissue biomechanics vary from site to site in both this porcine model and in human tissue are necessary to fully understand whether these findings have implications for surgical technique.

### Limitations

There were a number of limitations associated with this study which may influence the clinical relevance of the findings. Firstly, in the human knee, in full extension, the patella contacts the suprapatellar fat pad, in this study, the fat pad was removed and the kinematics used gave only cartilage-on-cartilage contact. The model does therefore not replicate full extension (of a human knee) and gives contact of the femoral trochlea equivalent to approximately 20 and 60 degrees flexion in a human joint [35]. In this model, the contact on the patella was larger than for a human knee with more superior contact than would occur in a human through the flexion range investigated [35], the differences between this PFJ model and a human knee may be as a result of using porcine tissue and may be exaggerated by the fixed flexion axis of the simulator. The removal of all soft tissue limited not only the contact with the suprapatellar fat pad but removed all ligaments which would particularly influence the medial-lateral constraint of the patella which in this study was unconstrained and relied on the congruent articulating surfaces and reduced flexion angle to prevent dislocation. The input kinematics were based on those used in previous wear simulation studies of the human artificial PFJ, it is not known whether these are appropriate for use with porcine tissue however, the development of an animal model allows for a reliable source of tissue of a consistent quality and can be further developed to use human tissue. In addition, there are a number of limitations of the techniques used for assessment of wear, damage and deformation. Using cartilage grading gives a rapid technique however the subjectivity of the method means there is potential for inter user variability and when using the grading system, the most severe damage in any region is scored irrespective of the size of the damage so a single scratch running the length of the patella covering 3 zones would result in the same score as a single region of grade 3 damage. For the geometrical measurement of the femoral trochlea, as well as the sensitivity of the technique making assessment of low volume change difficult, it was not possible to differentiate between wear, deformation or damage therefore, when interpreting the data, both the geometric measurements and the cartilage grading score should be considered. Future studies will also consider microscopic assessment of the cartilage structure perhaps using histological techniques to better understand cartilage damage. The study investigated only a single graft, multiple grafts may be used for larger defects which are likely to be less stable. Lastly, these were short-term studies lasting only 3 hours hence representing the initial stability of the graft and damage to the articulating surfaces that would occur immediately following implantation, prior to any tissue regeneration or integration.

## Conclusion

A pre-clinical experimental simulation model of the porcine PFJ to predict wear of osteochondral interventions has been established, with initial proof of concept studies investigating the performance of osteochondral grafts immediately post implantation. Future studies will translate the method into the human joint and apply the simulation to current and future products for use for treatment of osteochondral defects and continue to investigate the boundaries of a range of variables such as surgical precision.
